# Endoscopic and videofluoroscopic evaluations of swallowing for dysphagia: A systematic review^☆^^☆☆^

**DOI:** 10.1016/j.bjorl.2025.101598

**Published:** 2025-04-10

**Authors:** Mario Augusto Ferrari de Castro, Rogério Aparecido Dedivitis, Leandro Luongo de Matos, José Carlos Baraúna, Luiz Paulo Kowalski, Kauê de Carvalho Moura, Daniel Herman Partezani

**Affiliations:** a Universidade Metropolitana de Santos, Departamento de Cirurgia de Cabeça e Pescoço, Santos, SP, Brazil; b Universidade de São Paulo, Departamento de Cirurgia de Cabeça e Pescoço, São Paulo, SP, Brazil; c Hospital Ana Costa, Departamento de Cirurgia de Cabeça e Pescoço, Santos, SP, Brazil

**Keywords:** Deglutition disorders, Head and neck neoplasms, Laryngoscopy

## Abstract

**Objective:**

This systematic review was performed to compare the diagnostic accuracy of FEES and VFSS in evaluating swallowing in adults with dysphagia.

**Methods:**

A search for articles published between January 1991 and March 2020 was carried out in the MEDLINE EMBASE, COCHRANE, SciELO, and LILACS electronic databases. Based on the eligibility criteria, six articles were included.

**Results:**

FEES demonstrated a higher ability to diagnose pharyngeal residue, penetration, and aspiration compared with VFSS, and slightly better performance in detecting premature spillage.

**Conclusion:**

There were no significant differences on the diagnostic performance of both tests. The choice of test should depend on availability, team experience, and patient’s preference.

**Level of evidence:**

Level III.

## Highlights

• FEES demonstrated a higher ability to diagnose pharyngeal residue, penetration, and aspiration compared with VFSS.
• This exam was slightly better performance in detecting premature spillage.
• There were no significant differences on the diagnostic performance of both tests.
• The choice of test should depend on availability, team experience, and patient’s preference.

## Introduction

Up to 16% of the general population may experience dysphagia during lifetime,^1^ which can result from a variety of medical etiologies, including stroke, other neurologic conditions, and head and neck cancer.^2^ This condition heightens the risk of aspiration, leading to increased morbidity, impaired quality of life, and high risk of mortality.^3^, ^4^, ^5^, ^6^, ^7^

Early detection of dysphagia is essential to prevent adverse health outcomes. Fiberoptic Endoscopic Evaluation of Swallowing (FEES) and Videofluoroscopic Swallowing Study (VFSS) are widely used examinations for studying swallowing disorders. Their primary purpose is twofold: to identify and interpret the nature of the swallowing problem, and to guide therapeutic and rehabilitation interventions.^8^

While VFSS is still considered the gold standard in some studies,^9^, ^10^, ^11^, ^12^, ^13^, ^14^ numerous reports in the literature emphasize the validity of FEES because of its availability, patient compliance, and the expertise it requires. Recent reproducibility studies have found similar results for both tests.^15^, ^16^, ^17^, ^18^

This systematic review was conducted to compare the diagnostic accuracy of FEES and VFSS in detecting alterations in swallowing among adults with dysphagia.

## Methods

This research was approved by the Institutional Review Board of the Medicine School of University of São Paulo, under protocol nº 645.707, on May 13, 2014.

A systematic search for articles published between January 1991 and March 2020 was carried out in the MEDLINE, EMBASE, COCHRANE, SciELO, and LILACS electronic databases. A wide search strategy was employed to minimize publication bias. The following descriptors were used: (endoscopy OR fibroscopy) OR nasofibroscopy) OR Laryngoscopy) OR fibreoptic endoscopic) AND (videofluoroscopy or fluoroscopy). Exclusion criteria included: inability to obtain individual data, review articles, case reports, duplicate samples, and studies including individuals aged <18 years.

**Fig. 1:**
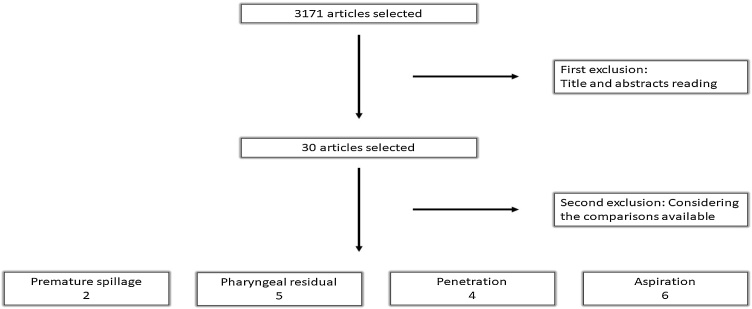
PRISMA flow diagram illustrating the systematic search results.

Two researchers independently extracted data from the studies using a standardized form. Initially, 3,171 abstracts were identified. After applying the established criteria and reviewing titles and abstracts, 30 articles were shortlisted. Upon reading the 30 articles in full, only six met the inclusion criteria. Findings were reported according to the PRISMA guidelines^19^, ^20^ (Fig. 1).

For statistical analyses, the Meta-DiScW Program (Clinical BioStatistics Unit – Hospital Ramón y Cajal, Madrid, Spain) was employed.^21^ Sensitivity and specificity values, positive and negative likelihood ratios, as along with their Confidence Intervals (95% CI), were calculated for each study individually. The diagnostic Odds Ratio (dOR) was also calculated. The dOR serves as an additional measure to gauge test accuracy, signifying the increased likelihood of achieving a correct diagnosis when the test is positive as opposed to when it is negative.

Complementarily, a Receiver Operating Characteristic (ROC) analysis was conducted, and the areas under the Summary (SROC) curves were calculated. This method diverges from conventional ROC analysis, which compares test accuracy across different positivity thresholds. In an SROC graph, each data point comes from a distinct study; however, diagnostic thresholds should be consistent across studies to prevent influencing the curve’s shape.^22^

## Results

Six studies were selected, comprising a total of × patients with stroke as the main cause of dysphagia (Table 1).

**Table 1:** Number of studies, patients, and their distributions by dysphagia etiology.

Author	Year	n	Gender F/M	Age (mean)	Stroke	Neurological diseases	Carotid bypass	Trauma	Malignant tumor	Others
Langmore et al.^23^	1991	21	21/0	63	9	6	1	0	1	4
Wu et al.^24^	1997	28	17/11	64.7	22	1	0	1	4	0
Périé et al.^25^	1999	7	2/5	50.6	0	0	1	0	6	0
Singh et al.^26^	2009	100	63/37	(19–100)	47	38	0	5	0	10
Rao et al.^21^	2010	11	9/2	50	3	3	0	2	0	3
Park et al.^22^	2015	50	31/19	67.8	32	6	0	3	5	4

Table 2 describes the sensitivity and specificity values, Positive Likelihood Ratio (PLR), and Negative Likelihood Ratio (NLR) of FEES compared to VFSS for detecting swallowing changes. FEES demonstrated superior diagnostic capability for pharyngeal residue, penetration, and aspiration compared to VFSS, with a moderate performance in detecting premature spillage.

**Table 2:** Sensitivity, specificity, Positive Likelihood Ratio (PLR), Negative Likelihood Ratio (NLR) by swallowing alterations comparing FESS and VFSS.

	Sensitivity^a^	Specificity^a^	PLR^a^	NLR^a^
Premature spillage	0.607	0.667	1.790	0.614
	(0.406‒0.785)	(0.430‒0.854)	(0.938–3.416)	(0.364–1.040)
Pharyngeal residue	0.966	0.591	2.376	0.091
	0.916‒0.991)	(0.485‒0.692)	(1.100–5.132)	(0.037‒0.291)
Penetration	1.000	0.832	3.884	0.076
	(0.923–1.000)	(0.762‒0.888)	(1.897–7.953)	(0.020‒0.292)
Aspiration	0.800	0.917	6.759	0.284
	(0.663‒0.900)	(0.874‒0.948)	(2.454–18.621)	(0.173‒0.465)

FEES, Fiberoptic Endoscopic Evaluation of Swallowing; VFSS, Videofluoroscopy.

a Value (95% CI).

**Fig. 2:**
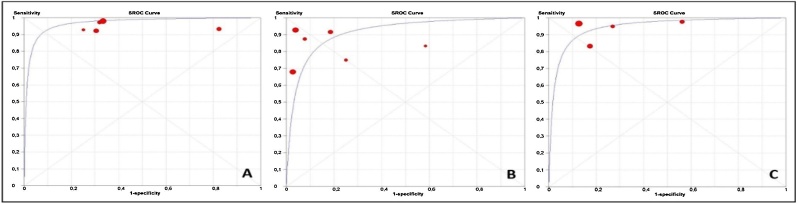
(A) Summary ROC (SROC) Curves comparing Fiberoptic Endoscopic Evaluation of Swallowing (FEES) and Videofluoroscopy (VFSS) for the diagnosis of pharyngeal residue. (B) Aspiration. (C) Penetration. The area under the ROC curve is 96.8%, 91.5% and 94.6%, respectively, for pharyngeal residue, aspiration, and penetration. Note: An SROC curve for premature spillage was not generated because of the availability of only two valid studies.

The diagnostic accuracy of FEES compared to VFSS for swallowing alterations is detailed in Table 3. FEES showed higher accuracy in diagnosing pharyngeal residue, penetration, and aspiration than VFSS. ROC analysis for premature spillage was not performed because of the availability of only two valid studies. Similarly, results from the dOR should be interpreted with caution for the same reason. The summary ROC curves are shown in Fig. 2.

**Table 3:** Area Under the ROC Curve (AUC) and diagnostic Odds Ratio (dOR) by swallowing alterations comparing FESS and VFSS.

	AUC^a^	Diagnostic dOR^b^
Premature spillage	‒	3.307 (0.971–11.264)
Pharyngeal residue	0.9678 (0.052)	28.983 (8.110–103.46)
Penetration	0.9457 (0.050)	56.480 (12.250–260.41)
Aspiration	0.9148 (0.035)	45.344 (4.476–142.04)

‒, Test not performed (only 2 valid studies); FEES, Fiberoptic Endoscopic Evaluation of Swallowing; VFSS, Videofluoroscopy.

a Value (standard error).

b Value (95% CI).

## Discussion

FEES and VFSS are considered the best tests for objectively evaluating oropharyngeal dysphagia.^8^, ^11^, ^27^, ^28^

VFSS, often termed the “reference standard”, is a frequently embraced instrumental evaluation for dysphagia, as it provides comprehensive information regarding anatomical and physiological functions, aiding in both diagnosis and treatment planning.^14^ The main advantages of VFSS in relation to other swallowing assessment methods include the integrated observation of all swallowing phases, which encompasses the oral preparatory and transit phases, the elevation and anterior displacement of the hyoid-larynx complex, the opening of the upper esophageal sphincter, and esophageal transit.^29^

Conversely, the advantages of FEES are its potential use in cases with limited assessment by VFSS (outside the radiology suite, of patients with limiting postural problems, and of patients at great risk for laryngotracheal aspiration during VFSS).^30^ Given constraints like availability, patient compliance, obesity, need for bedside exams, and specific expertise, VFSS may not be feasible for every patient suspected of dysphagia.^14^

However, experts disagree about which of these tests should be considered the gold standard for assessing oropharyngeal dysphagia. Some are in favor of VFSS,^31^, ^32^, ^33^, ^34^ whereas others believe that both tests merit this designation.^8^, ^11^, ^35^ There are four technical limitations concerning VFSS: (1) Radiation exposure; (2) Uncooperative patients, especially those with postural or emotional limitations; (3) Preparation of the physical structure and materials, in addition to patient transportation; and (4) Limited capability for an in-depth investigation of anatomical anomalies.^36^ Echoing our findings, recent reproducibility studies have also reported similar results for both tests.^5^, ^18^

Our results support the view that both FEES and VFSS are invaluable procedures for evaluating dysphagia. Notably, we found that FEES presents higher efficacy in diagnosing pharyngeal residue, penetration, and aspiration.

Since VFSS and FEES are statistically comparable, both deserve to be considered as gold standards. The choice of instrument should hinge on clinical indications, equipment availability, and evaluators’ clinical expertise. Furthermore, it is important that clinicians recognize the strengths and weaknesses of each diagnostic procedure. For instance, while VFSS provides greater information during the oral phase of swallowing, it can be impracticable for certain groups of patients. In contrast, FEES can provide the examiner with additional information on the anatomy and physiology of the pharynx and larynx that the VFSS could not. Ideally, VFSS and FEES should be used to complement each other.

A limitation of this study is the heterogeneous design of the primary articles, which comprises two retrospective and four prospective studies. A particular difficulty was the absence of a unanimous gold standard method for evaluating dysphagia in our patient cohort.

## Conclusion

This systematic review indicates that both FEES and VFSS are effective for instrumental assessment of swallowing in patients. The differences between the two tests are not statistically significant. Therefore, the optimal test should be chosen based on the examination location, equipment availability, expertise of the team, and patient preferences.

## Meeting of ethical standards

O presente artigo faz parte da coleção de artigos que anteriormente pertenciam a Sociedade de Cabeça e Pescoço (SCCP) e foram cedidos ao Brazilian Jornal of Otorhinolaryngology (BJORL).

## Declaration of competing interest

The authors declare no conflicts of interest.
